# Infographic: the Pneumatic Retinopexy versus Vitrectomy for the Management of Primary Rhegmatogenous Retinal Detachment Outcomes Randomized Trial (PIVOT)

**DOI:** 10.1038/s41433-021-01597-9

**Published:** 2021-06-30

**Authors:** Declan C. Murphy, Nikolaos Tzoumas, Alex Mehta, Islam Mostafa, Salman N. Sadiq, Anna Song, Mo Al-Zubaidy, Ali E. Ghareeb, David H. Steel

**Affiliations:** Biosciences Institute, Newcastle University, Newcastle upon Tyne, UK

**Keywords:** Education, Surgery

**Reference**: Hillier RJ, Felfeli T, Berger AR, Wong DT, Altomare F, Dai D, et al. The Pneumatic Retinopexy versus Vitrectomy for the Management of Primary Rhegmatogenous Retinal Detachment Outcomes Randomized Trial (PIVOT). Ophthalmology. 2019;126:531–9. 10.1016/j.ophtha.2018.11.014.

**Fig. 1:**
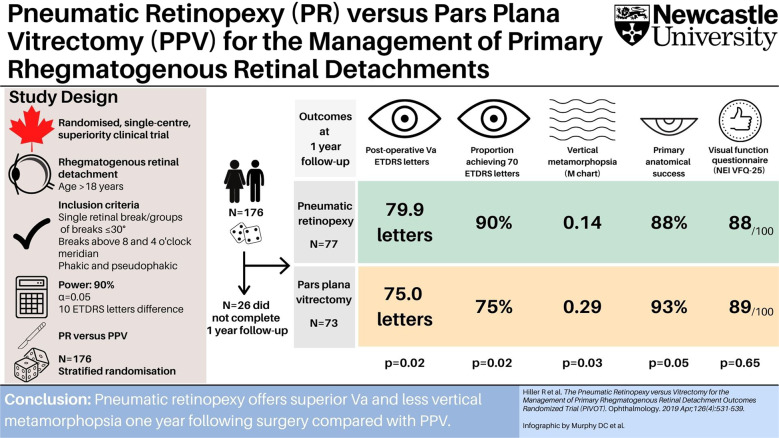
The Pneumatic Retinopexy versus Vitrectomy for the Management of Primary Rhegmatogenous Retinal Detachment Outcomes Randomized Trial (PIVOT) showed that at 1 year following surgery, pneumatic retinopexy achieved superior outcomes in terms of visual acuity and vertical metamorphopsia. There was no significant difference in patient-reported subjective visual function between the two surgical interventions. Abbreviations: ETDRS Early Treatment Diabetic Retinopathy Study, NEI VFQ-25 questionnaire National Eye Institute Visual Function Questionnaire, PPV pars plana vitrectomy, PR pneumatic retinopexy, Va visual acuity.

